# Primary Jejunal Impactions Resolved via Exploratory Celiotomy in Six Horses: 2017–2023

**DOI:** 10.3390/ani15162363

**Published:** 2025-08-12

**Authors:** Jaclyn Willette, Alyssa Guinn, Amelia Munsterman

**Affiliations:** 1 Department of Veterinary Clinical Sciences, Iowa State College of Veterinary Medicine, Ames, IA 50011, USA; 2 Department of Large Animal Clinical Sciences, College of Veterinary Medicine, Michigan State University, East Lansing, MI 48824, USA

**Keywords:** equine, jejunum, colic, impaction

## Abstract

Impactions of the jejunum are rarely described in the literature. The current case series describes six cases of adult horses with jejunal impactions with feed material diagnosed by exploratory celiotomy. Horses underwent exploratory celiotomy based off of their degree of pain despite medical management and concerns for a primary strangulating small intestinal lesion. All jejunal impactions were relieved via manual decompression of the impaction into the cecum. None of the cases underwent a resection or anastomosis at the site of impaction. All horses were treated with gastroprotectants (omeprazole or sucralfate) post-operatively; 3/6 horses underwent a gastroscopy and were diagnosed with squamous gastric ulceration prior to treatment. Despite post-operative complications, 5/6 horses survived to hospital discharge.

## Simple Summary

To the author’s knowledge, primary jejunal impactions requiring exploratory celiotomy for resolution have not been reported in the literature. The current case series describes six cases of adult horses diagnosed with jejunal impactions and managed surgically via exploratory celiotomy and manual decompression. None of the cases required an enterotomy or resection and anastomosis at the impaction site. Post-operatively all horses were treated with gastroprotectants (omeprazole or sucralfate) along with standard post-operative medical management. Three of the six horses underwent a gastroscopy and were diagnosed with squamous gastric ulceration prior to treatment. Despite post-operative complications, 5/6 horses survived to hospital discharge.

## 1. Introduction

Small intestinal strangulating lesions in horses are the most common cause of surgical small intestinal lesions, with the remainder diagnosed as non-strangulating either simple or functional obstructions [1,2,3,4]. Common non-strangulating lesions include ileal impaction, ileal muscular hypertrophy, and proximal enteritis [5,6,7]. Intraluminal obstruction of the small intestine has been reported to be caused by impacted feed material, foreign bodies including phytotrichobezoars or enteroliths, and ascarids in juveniles [8,9].

An intestinal impaction in horses is defined as an obstruction typically caused by dehydrated intraluminal ingesta or non-feed materials including sand or gravel. Impactions typically occur at sites where there is transition of intestinal movement due to physiologic or anatomic pacemaker activity, at intestinal sphincters, or where the lumen narrows naturally or secondary to intestinal adhesions. Other causes may include mesenteric thrombosis which alter motility or mural diverticulum [10]. Reported sites of predilection for impactions in horses include the cecum, pelvic flexure, right dorsal colon, and ileum.

Impactions of the jejunum are rarely described in the literature. Causes previously reported have focused on foreign bodies, including molasses feed blocks, baling twine, persimmon fruit phytobezoars as well as neoplasia. Feed material causing intraluminal obstruction rarely has been considered a differential for a primary small intestinal surgical obstruction [3,11]. This retrospective case series aims to describe the clinical presentation and surgical treatment of horses with primary jejunal feed impactions. To the author’s knowledge this is the first case series describing this lesion as the primary reason for colic requiring resolution via exploratory celiotomy.

## 2. Case Histories and Clinical Findings

### 2.1. CASE 1

A 9-year-old Oldenburg gelding was referred for an acute history of severe colic that was unresponsive to medical management at a horse show. The gelding originally resided in Kentucky and arrived in Michigan 3 days prior to the episode of colic.

Admission vital parameters revealed a heart rate of 52 bpm, respiratory rate of 22 brpm, and a rectal temperature of 36.9 °C (98.5 °F). Cardiac auscultation revealed a grade II/VI systolic murmur, with intermittent 2nd degree AV block. Mucous membranes were pink and tacky, with a capillary refill time of 2 s and abdominal auscultation revealed decreased borborygmi in all abdominal quadrants. Packed cell volume was 43% and total solids was 6.5 g/dL. Venous blood gas revealed a mild hyperlactatemia at 0.9 mmol/L (RR: <0.7 mmol/L). Nasogastric intubation yielded 11 L of net reflux and the tube was left indwelling for further gastric decompression. Transcutaneous abdominal and thoracic ultrasonography revealed multiple populations of distended small intestine (approximately 5 cm in diameter) imaged bilaterally in the inguinal region. The remainder of the admission abdominal and thoracic ultrasonographic evaluation was unremarkable. Abdominal palpation per rectum revealed marked distension of several loops of small intestine that were not compressible upon palpation. Abdominocentesis yielded clear yellow fluid with an L-lactate of 2.6 mmol/L and a total solids of less than 2 g/dL. A full summary of admission clinical data can be found in Table 1.

The gelding was refractory to medical management, including intravenous fluids and analgesia, prompting recommendations for an exploratory celiotomy. Exploratory celiotomy revealed a 60 cm mid-jejunal impaction of firm ingesta. No palpable intraluminal foreign bodies were noted. The impaction was relieved via manual decompression utilizing carboxymethylcellulose of the small intestines into the cecum. Additional findings included gastric distention with liquid ingesta despite the presence of an indwelling nasogastric tube. Firm desiccated digesta was noted in the large colon and a pelvic flexure enterotomy was performed routinely to evacuate the contents. The remainder of the abdominal exploration was unremarkable.

### 2.2. CASE 2

A 21-year-old Quarter Horse gelding was referred for treatment of severe colic and a suspected strangulating small intestinal surgical lesion. Admission vital parameters revealed a heart rate of 64 bpm, respiratory rate of 18 brpm, and a rectal temperature of 37.7 °C (99.9 F). Mucus membranes were pink and tacky, with a capillary refill time of 3 s. No abnormalities were noted on thoracic auscultation and abdominal auscultation revealed decreased borborygmi in all abdominal quadrants. Packed cell volume was 37% and total solids was 7.1 g/dL with a hyperlactatemia of 7.8 mmol/L (RR: <0.7 mmol/L). The remainder of the admission venous blood gas can be found in Table 1. Nasogastric intubation yielded 15 L of spontaneous net reflux. Transcutaneous thoracic and abdominal ultrasonography revealed no abnormalities in the thorax and dilated (diameter of 6–7 cm), amotile loops of small intestine diffusely throughout the inguinal regions. Abdominal palpation per rectum revealed dilated loops of small intestine. Abdominocentesis yielded dark yellow peritoneal fluid with an L-lactate of 12.7 mmol/L, total solids of 2.8 g/dL, and a glucose of 227 mg/dL.

Based on the admission clinical data, exploratory celiotomy was recommended due to concerns of a strangulating small intestinal lesion. Exploratory celiotomy revealed a mid-jejunal impaction of approximately 1.8 m of mid-jejunum. The impaction was resolved via manual decompression into the cecum utilizing carboxymethylcellulose. No further lesions were identified on abdominal exploration. The area of impaction was edematous and red, with mesenteric hemorrhage secondary to the marked bowel distension; however, the bowel was deemed viable, and no resection and anastomosis was performed.

### 2.3. CASE 3

A 15-year-old Morgan mare was referred following an episode of severe colic. The mare had a history of laminitis, and 8 episodes of colic that were resolved medically on the farm in the previous year. Previous diagnostics included gastroscopy by her primary care veterinarian, which was clinically unremarkable. The mare was maintained intermittently on oral phenylbutazone (1 g orally every 12 h) or firocoxib (Equioxx 0.09 mg/kg orally once daily) for management of her laminitis and treated empirically for pituitary pars intermedia dysfunction (PPID) with 1 mg of oral once daily pergolide (Prascend).

Vital parameters upon presentation revealed a tachycardia at 60 bpm, a respiratory rate of 22 brpm, and a rectal temperature of 38.0 °C (100.5 °F). Mucus membranes were pink and moist, with a capillary refill time of less than 2 s. Gastrointestinal borborygmi were decreased in all abdominal quadrants and cardiothoracic auscultation was unremarkable. Digital pulses were elevated on all limbs and the mare was sensitive to hoof testers on presentation.

Packed cell volume was 42% and total solids was 7.3 g/dL with a mild hyperlactatemia of 2.4 mmol/L (RR: <0.7 mmol/L). The remainder of the admission venous blood gas can be found in Table 1. Nasogastric intubation yielded 5 L of net reflux. Transcutaneous abdominal and thoracic ultrasonography revealed dilated hypomotile small intestines in the left inguinal region. Abdominal palpation per rectum revealed a tight band of the presumptive large colon coursing transversely across the caudal abdomen indicative of a suspected right dorsal large colonic displacement. Abdominocentesis yielded dark yellow peritoneal fluid with an L-lactate of 7.3 mmol/L, a total solids of 2.6 g/dL, and a glucose of 147 mg/dL.

Medical management was initially recommended and the mare was started on intravenous fluid therapy with lactated Ringer’s solution at 120 mL/kg/day, with intermittent gastric decompression via the indwelling nasogastric tube. The mare was administered a 1.1 mg/kg IV dose of flunixin meglumine. However, the mare became progressively more painful prompting recommendations for an exploratory celiotomy.

Exploratory celiotomy revealed peritonitis, with moderate amounts of free fibrin in the abdomen. In addition, an approximately 1.8 m, mid-jejunal impaction was noted, composed of fibrous feed material and palpable grit consistent with sand. Oral to the jejunal impaction, the jejunum was moderated distended with gas and fluid. The impacted jejunum was diffusely hyperemic, with moderate amounts of serosal and mesenteric hemorrhage. Additionally, a right dorsal displacement of the large colon was noted.

The mid-jejunal impaction was manually decompressed into the cecum utilizing carboxymethylcellulose lavage to resolve the impaction, and the right dorsal displacement of the large colon was corrected. The remainder of the abdominal exploration was unremarkable. A punch biopsy was obtained from the mid-jejunum and closed using a cruciate suture pattern with 2–0 poliglecaprone 25 and oversewn with a Lembert pattern with 2–0 poliglecaprone 25.

### 2.4. CASE 4

A 12-year-old Thoroughbred gelding was referred for severe signs of acute colic. The gelding had no prior history of colic before this episode and was at a horse show at the time clinical signs were noted. Vital parameters on presentation revealed a tachycardia at 64 bpm, a respiratory rate of 20 brpm, and a rectal temperature of 37.8 °C (100.1 °F). Mucus membranes dark pink and tacky, with a capillary refill time of 3–4 s. Cardiothoracic auscultation was unremarkable, and the gelding had reduced jugular refill bilaterally. Gastrointestinal auscultation revealed hypermotile borborygmi in the left dorsal quadrant and absent borborygmi in the remaining gastrointestinal quadrants.

Packed cell volume was 55% and total solids was 9 g/dL with a mild to moderate hyperlactatemia of 3.1 mmol/L (RR: <0.7 mmol/L). The remainder of the admission venous blood gas can be found in Table 1. Nasogastric intubation yielded approximately 10 L of malodorous, brown-tinged net reflux.

Transcutaneous abdominal and thoracic ultrasonography revealed a normal appearance to the left and right kidney, spleen, and liver. The greater curvature of the stomach was noted at the 15th intercostal space. The diameter, wall thickness, motility, and location of the small and large intestine were within normal limits. There was no significant increase in free peritoneal fluid. Brief thoracic and cardiac evaluation was unremarkable. Abdominal palpation per rectum yielded a small pile of mucus-covered manure in the rectum. Two loops of turgid, distended small intestines were palpable in the caudoventral abdomen. Abdominocentesis yielded dark yellow peritoneal fluid with an L-lactate at 3.2 mmol/L, a total solids of 4 g/dL, and a glucose of 228 mg/dL.

Based on the gelding’s initial examination and diagnostics, and due to concerns for a strangulating lesion, exploratory celiotomy was recommended. Exploratory celiotomy revealed an approximately 3 m, desiccated feed impaction of the distal jejunum. The small intestine oral to obstruction was distended and petechia resulted from light manipulation. The small intestine distal to the obstruction was pink and collapsed. The intestines were deemed viable, and no resection and anastomosis was performed. The impaction was resolved via manual decompression into the cecum utilizing carboxymethylcellulose. The remainder of the exploration was unremarkable.

### 2.5. CASE 5

A 6-year-old Quarter Horse gelding was referred for a 24 h history of progressively worsening colic signs that were unable to be managed while at a horse show. He was originally diagnosed and managed as a suspect pelvic flexure impaction; however, his colic signs progressed prompting referral. Vital parameters upon presentation revealed a mild tachycardia at 44 bpm, a respiratory rate of 12 brpm, and a rectal temperature of 36.8 °C (99.5 °F). Mucus membranes were pale, pink and tacky, with a capillary refill time of 2–3 s. Cardiothoracic auscultation was unremarkable. Gastrointestinal borborygmi were decreased in all abdominal quadrants.

Packed cell volume was 37% and total solids was 5.5 g/dL with a mild hyperlactatemia of 2.3 mmol/L (RR: <0.7 mmol/L). The remainder of the admission venous blood gas can be found in Table 1. Nasogastric intubation yielded approximately 7 L of net reflux. Transcutaneous abdominal and thoracic ultrasonography revealed no abnormalities in the thorax and dilated colonic vessels on the right side of abdomen. Abdominal palpation per rectum revealed small, hard fecal balls coated in mucus in the rectum and the pelvic flexure was gas distended with a tight colonic band coursing to the right side of the caudal abdomen. Abdominocentesis yielded clear yellow peritoneal fluid with an L-lactate of 2.6 mmol/L and a total solids of less than 2 g/dL. Medical management was initially elected. However, the horse became acutely and severely painful once he was placed in a stall and was refractory to sedation prompting recommendations for exploratory celiotomy.

Exploratory celiotomy revealed several fibrous adhesions spanning from the right caudal abdominal body wall to the cecum and from the caudal ventral midline of the abdominal body wall to the large colon causing significant traction on the large colon and cecum. Several caudodorsal abdominal adhesions were present and the large colon had passed through some of these adhesions causing tension on the adhesions and gas distension of the large colon. Further abdominal exploration revealed a distal jejunal feed impaction of ~2.4 m. The jejunal impaction was resolved by manual decompression into the cecum utilizing carboxymethylcellulose. Manual adhesiolysis was performed by blunt dissection for several adhesions between the cecum and ventral body wall. The large colon was then exteriorized and desiccated digesta was noted within the right dorsal and right ventral colon. The caudodorsal fibrous adhesions had palpable arterial pulsations preventing blind adhesiolysis and therefore were left intact. A pelvic flexure enterotomy was performed to evacuate the large colon impaction routinely. The remainder of the abdominal exploration was unremarkable.

### 2.6. CASE 6

An 11-year-old Oldenburg gelding was referred for treatment of moderate to severe colic signs unresponsive to medical management on the farm. The gelding was at a horse show throughout the weekend and had shipped home a few days prior to the onset of his colic signs. Prior to presentation, the gelding displayed mild signs of colic initially managed with oral flunixin meglumine and enteral fluid therapy on the farm. However, he became progressively more uncomfortable prompting referral. Vital parameters on presentation revealed a tachycardia at 50 bpm, a respiratory rate of 20 brpm, and a rectal temperature of 37.6 °C (99.7 °F). Mucous membranes were pale pink and tacky, with a capillary refill time of greater than 3 s. Cardiothoracic auscultation was unremarkable. Gastrointestinal borborygmi were decreased in all abdominal quadrants.

Packed cell volume was 33% and total solids was 7.1 g/dL with a mild hyperlactatemia of 1.1 mmol/L (RR: <0.7 mmol/L). The remainder of the admission venous blood gas can be found in Table 1. Nasogastric intubation yielded approximately 2 L of net reflux. Transcutaneous abdominal and thoracic ultrasonography revealed several dilated (up to 5–6 cm) amotile small intestinal loops with sedimented ingesta and normal wall thickness. Abdominal palpation per rectum revealed moderately firm ingesta in the pelvic flexure in the left caudal ventral abdomen and several turgid 5–6 cm loops of gas distended non-compressible small intestinal loops palpable in the caudal right abdomen. Abdominocentesis yielded bright yellow peritoneal fluid with an L-lactate of 3.1 mmol/L, a total solids of 2.6 g/dL, and a glucose 127 mg/dL.

**Figure 1:**
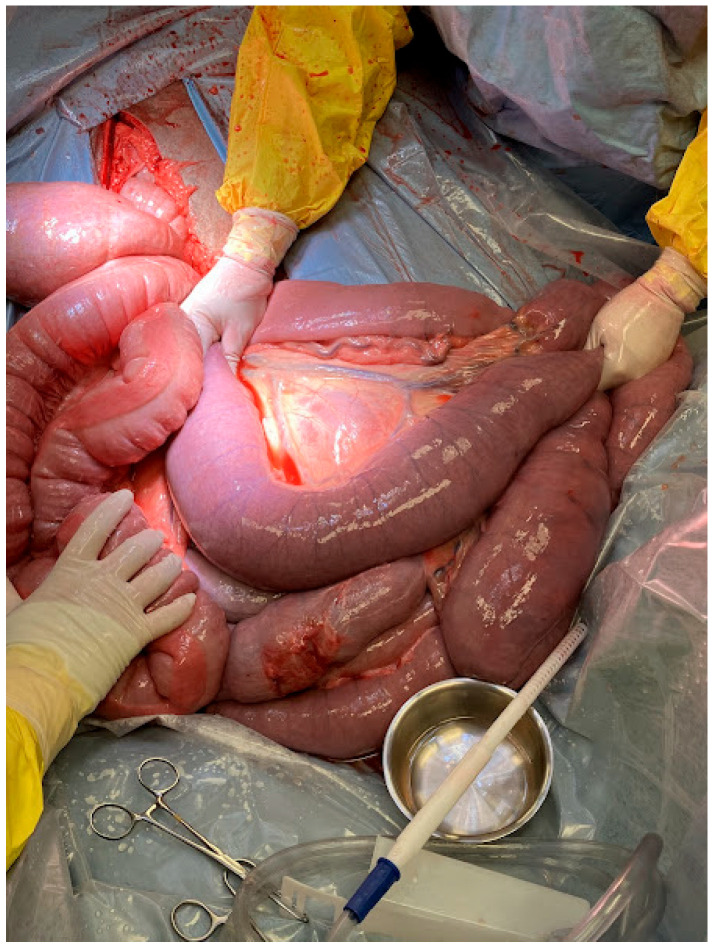
Intraoperative picture of case 6 showing the distal jejunal impaction between the surgeons’ hands.

Due to persistent pain refractory to medical management and concerns for a primary small intestinal lesion, exploratory celiotomy was recommended and elected. Exploratory celiotomy revealed an impaction of the distal jejunum, with significant gas distension of the oral small intestine (Figure 1). The impaction was easily resolved with extraluminal manipulation and decompressed into the cecum utilizing carboxymethylcellulose. There was mild ecchymoses in the oral jejunum secondary to prolonged distention, but the bowel was deemed viable. The large colon contained desiccated ingesta, and the cecum was fluid-filled following decompression of the small intestine. The remainder of the exploration was unremarkable.

## 3. Outcomes

All horses underwent routine abdominal closures and recovered without incident from general anesthesia. All horses were administered peri-operative antimicrobials consisting of potassium penicillin (22,000 U/kg Q6) and gentamicin (6.6 mg/kg Q24). Pain management was provided with flunixin meglumine (1.1 mg/kg Q12 initially) as a non-steroidal anti-inflammatory, and they were administered continuous rate infusions of lidocaine (1.3 mg/kg bolus, followed by 0.05 mg/kg/min) and maintained on lactated Ringer’s solution intravenously for fluid maintenance post-operatively.

Case 1 was diagnosed 48 h after surgery with ileus and post-operative reflux requiring nasogastric intubation and gastric decompression every 2 h. Serum chemistry 48 h post-operatively revealed a mild azotemia with a creatinine of 1.8 mg/dL (reference range (RR): 0.9–1.6), hyperglycemia 176 mg/dL (RR: 77–105), increased total bilirubin 7.1 mg/dL (RR: 0.6–1.9), increased direct bilirubin 0.7 mg/dL (RR: 0.5–1.4), and increased triglycerides 395 mg/dL (RR: 10–42). A continuous rate infusion of metoclopramide (0.04 mg/kg/hour IV) was initiated in conjunction with the lidocaine continuous rate infusion. Additionally, the gelding’s antimicrobials were empirically switched to ceftiofur sodium (2.2 mg/kg IV every 12 h) and metronidazole (15 mg/kg orally every 8 h). A continuous rate infusion of dextrose, potassium chloride, and amino acids (partial parenteral nutrition) was initiated to provide parenteral nutrition. Additionally, he was administered omeprazole (1 mg/kg orally once daily) as a gastroprotectant. Despite intensive medical management, the gelding deteriorated and 6 days post-operatively became acutely painful prompting repeat exploratory celiotomy.

Exploratory celiotomy revealed small intestinal gas distention and a mid-jejunal small intestinal volvulus and a right dorsal large colonic displacement. The volvulus and displacement were manually corrected and the remainder of the abdominal explore was unremarkable. Anesthetic recovery was uneventful. Post-operatively following the second surgery, the gelding developed an incisional infection. Culture and susceptibility yielded growth of numerous *Klebsiella pneumoniae*, *Enterobacter cloacae*, and *Pseudomonas aeruginosa* with susceptibility to enrofloxacin. The gelding was provided a 14-day course of oral enrofloxacin and via ultrasonographic guidance incisional drainage was established. The gelding was maintained in an abdominal bandage (CM Heal Hernia Belt; CM Equine Products) for support. Case 1 was discharged from the hospital after a total of 15 days of hospitalization.

Case 2 recovered from surgery without incident and tolerated refeeding adequately. No complications were noted for the duration of his hospitalization. The only additional medication he was provided was omeprazole (1 mg/kg orally once daily) as a gastroprotectant. The gelding was discharged after 6 days of hospitalization.

Jejunal biopsy results for case 3 revealed mild, acute, suppurative enteritis. The karyorrhectic debris indicated some level of tissue hypoxia, but the tissue was not infarcted. Post-operatively case 3 developed ileus and post-operative reflux requiring intermittent gastric decompression until ~96 h post-operatively. Post-operative foot radiographs revealed no acute changes to the hoof capsule. Treatment with gabapentin (10 mg/kg orally every 8 h) was initiated for chronic foot pain. Serum chemistry 96 h post-operatively revealed a mild azotemia with a creatinine of 2.1 mg/dL (RR: 0.9–1.6) and a BUN of 40 mg/dL (RR:11–23 mg/dL). Due to a history of prolonged administration of non-steroidal anti-inflammatories and recurrent colic, gastroscopy was performed and noted moderate grade 2 ulceration of the squamous gastric mucosa. The mare was started on oral sucralfate (20 mg/kg orally every 8 to 12 h) and omeprazole (1 mg/kg orally every 24 h). The mare tolerated refeeding following resolution of her post-operative reflux; however, she remained hyporexic at the time of hospital discharge 8 days post-operatively.

Case 4 initially recovered well post operatively and after 24 h his pro-motility agents (continuous rate infusions of lidocaine 50 mcg/kg/min and metoclopramide 0.02 mg/kg/h) were discontinued. Two days post-operatively case 4 became inappetent and repeat transcutaneous abdominal ultrasound indicated gastric distention and hypomotile distended small intestine indicative of post-operative ileus. A nasogastric tube was passed, 10 L of net reflux was obtained and the tube was left indwelling for intermittent gastric decompression. Prokinetics (metoclopramide and lidocaine) were re-started.

Four days post-operatively, the indwelling nasogastric tube was removed following cessation of post-operative reflux. Approximately 12 h later case 4 became acutely colicky again prompting attempts at replacing the nasogastric tube which was unsuccessful. Esophagoscopy revealed severe pharyngitis and ulceration of the cranial esophageal sphincter precluding replacement of the nasogastric tube. The stomach was decompressed via the biopsy channel. The following morning case 4 underwent an esophagostomy under standing sedation. He was administered sucralfate (20 mg/kg orally every 8 h) for ulceration management via the indwelling esophagostomy tube.

Two days following placement of the esophagostomy tube, case 4 became acutely painful despite gastric decompression. Gastroscopy revealed grade 2 squamous ulceration but did not identify a pyloric outflow obstruction. Normal pyloric motility was observed. The gelding continued to decline and remained persistently painful. Repeat celiotomy was recommended and declined. Humane euthanasia was elected 12 days post-operatively. Post-mortem examination revealed mesenteric adhesions aboral to the duodenocolic ligament causing a functional partial obstruction.

Case 5 recovered from surgery and tolerated refeeding without incident. No complications were noted for the duration of his hospitalization. Per owner request he was prophylactically started on omeprazole (0.2 mg/kg orally once daily) and sucralfate (20 mg/kg orally every 12 h); gastroscopy was declined at the time of hospitalization. Case 5 was discharged 5 days post-operatively with prescriptions for omeprazole and sucralfate.

Case 6 underwent a post-operative gastroscopy and was diagnosed with grade 3/4 ulcerations in the squamous portion of the stomach, with the majority occurring at the lesser curvature. Mild inflammation and two small nodules were noted at the pyloric antrum in the glandular portion of the stomach. The patient was treated with omeprazole (1 mg/kg orally once daily) and sucralfate (20 mg/kg orally every 12 h). The gelding tolerated refeeding adequately and exhibited no post-operative signs of colic. He was discharged 7 days post-operatively without incident and continued on omeprazole and sucralfate.

## 4. Discussion

Jejunal impactions have been reported to be associated with a predisposing factor leading to luminal obstruction such as a neoplastic lesions or an impaction of foreign material such as compressed cracked corn, trichophytobezoar, compacted wood fragments, persimmon fruit, molasses-based stable treats, baling twine, and choleliths [12,13,14,15,16,17,18,19,20]. This case series describes primary surgical lesions resulting from jejunal impactions of ingested feed, with no external diverticulum, adhesion or mural neoplasia. The lack of an initiating factor, anatomic abnormality or intraluminal foreign body is unique to these cases. Short-term survival was 83%.

All impactions were resolved via manual decompression of the impaction into the cecum, as the affected jejunum was viable. Case 3 and Case 5 had additional lesions noted at exploratory celiotomy. Case 3 was diagnosed with a right dorsal displacement in addition to the jejunal impaction. Case 5 had several intra-abdominal adhesions in addition to the jejunal impaction. It is hard to fully elucidate in these cases which lesion was the main contributor to the horses pain that was refractory to medical management. It is plausible that in Case 3 the right dorsal large colonic displacement was secondary to the jejunal impaction. In Case 5 the adhesions were likely chronic lesions and it is plausible that the jejunal impaction led to a short-term change in the horse’s comfort level necessitating surgical exploration. However, in Case 5, the adhesions could have predisposed the horse to the jejunal impaction and could represent a predisposing factor.

Approximately 50% of the horses developed post-operative reflux, one horse developed post-operative small intestinal volvulus, and half of the horses were diagnosed with gastric or esophageal ulcers by gastroscopy. Gastroscopy was not performed in all cases due to clinician discretion. It is unknown if the other horses who did not undergo a post-operative gastroscopy also suffered from gastric ulceration in the present case series.

While jejunal impactions are rare, ileal impactions are the most commonly diagnosed impaction involving the small intestines [21]. The exact cause has not been fully elucidated, but associations with fine, high-lignin forage, coastal Bermuda hay, and tapeworm infestation (*Anoplocephala perfoliata*) have been reported [5,22,23]. All of the horses in the present study were fed diets of twice daily concentrates of various brands and twice daily either mixed timothy or timothy alfalfa hay. All horses had variable access to pasture turn-out for parts of the day with stall rest at night. None of the horses were fed coastal Bermuda hay. Historical administration of praziquantel was not provided, but it would be assumed that parasitic infiltration of the ileocecal valve would cause impactions more distal than the jejunum. Therefore, the cause for jejunal impactions is likely unrelated to predisposing factors for ileal impactions.

Historically, the decision to undergo medical or surgical management for the treatment of ileal impactions when this is the presumptive diagnosis has been a difficult decision for practitioners. Diagnostic criteria such as admission clinical and physical examination parameters, including severity of abdominal pain, heart rate, presence or absence of borborygmi, abdominal palpation per rectum findings, and abdominal ultrasound examination findings, were inconclusive in directing the clinician to the ideal therapy for successful resolution of an ileal impaction [5]. In contrast, all horses in the current case series were moderate to markedly painful on or shortly following admission and refractory to initial pain management. Of the six horses, an exploratory celiotomy was recommended immediately following the admission examination for 2 of the cases due to concerns of a strangulating small intestinal lesion. Medical management was initially attempted for Cases 1, 3, 5, and 6; however, shortly following the initiation of medical management all cases were refractory prompting recommendations for abdominal exploration. The severity of pain was consistent in all cases with jejunal impactions.

The source of abdominal discomfort in ileal impactions is presumed to be associated with the spasmodic contraction of the intestine around the impaction. As the pain progresses in severity, there is a concomitant degree of jejunal distension oral to the impaction [5,8]. Medical management has been successfully used to treat ileal impactions, consisting of intravenous fluid therapy, enteral fluid therapy if no gastric reflux is obtained, and anti-inflammatory pain medications or other analgesics if indicated [5,8,22]. It is unknown if jejunal impactions can be treated medically, as they are difficult to identify based on clinical examination. A previous report noted that persistent pain and gastric reflux are more common in horses requiring exploratory celiotomy for treatment of ileal impactions, which may be consistent with the findings in the current case series [5]. In the same study, 1-year survival was 91% for horses diagnosed with ileal impactions and were treated by surgery and 92% for those horses treated for ileal impactions medically [5]. A more recent study reported lower survival rates (75.8–78.4%) for surgically treated ileal impactions [24]. Survival percentage of the current manuscript was 83.3% of horses diagnosed with jejunal impactions who underwent exploratory celiotomy.

Equine Squamous Gastric Disease or (ESGD) was diagnosed in cases 3,4 and 6 by gastroscopy in hospital and all horses were treated for gastric ulceration during hospitalization with either omeprazole (5/6 horses), sucralfate (4/6 horses) or both. ESGD describes lesions involving the squamous mucosa including the margo plicatus, greater and lesser curvatures, and the dorsal squamous fundus [25]. This can then be further classified into primary or secondary based on the known pathophysiology of disease. Primary ESGD occurs in an otherwise healthy gastrointestinal tract versus secondary ESGD is due to delayed gastric outflow as sequela to other diseases [25,26,27,28,29]. A previous study concluded that there is an association between gastritis and more distal small intestinal inflammation [30]. Horses with unclassified gastric disease may also have lower gastric and fecal microbial diversity compared to healthy controls [31]. There is some evidence to suggest an increased incidence of colic and post-prandial discomfort exists in these horses [26,29,32,33,34,35,36,37]. It is plausible that the presence of ESGD could be associated with jejunal impactions in horses as either a causative factor or resulting from intestinal ileus. From this retrospective case series, definitive conclusions on an association with horses diagnosed with ESGD potentially then developing a jejunal impaction cannot be concluded and would require further studies to validate this correlation.

A single fatality was observed in this case series, due to several post-operative complications that led to humane euthanasia. Post-mortem examination revealed mesenteric adhesions just aboral to the duodenocolic ligament resulting in a functional partial obstruction. This partial obstruction resulted in the post-operative colic and ileus noted ante-mortem. Case 5 also had several adhesions noted intra-abdominally at the time of exploratory celiotomy requiring adhesiolysis. Abdominal adhesion formation has previously been reported in 9% to 27% of horses undergoing repeat celiotomy or post-mortem examination [38,39,40]. Adhesions could be a risk of jejunal impactions, due to the intestinal distension caused by the impaction and ileus, along with the mechanical stress caused by decompression to resolve the impaction. Methods to reduce adhesion formation reported in the literature include appropriate antimicrobial and anti-inflammatory therapy, heparin administration, intraperitoneal sodium carboxymethylcellulose, sodium hyaluronate, intraperitoneal instillation of fucoidan solution, intraperitoneal crystalloid instillation, peritoneal lavage, and omentectomy [39,40,41,42,43,44,45,46,47,48,49,50,51,52,53,54,55]. All cases in the present manuscript were treated with broad-spectrum antimicrobials, anti-inflammatories, and intraperitoneal use of intraperitoneal sodium carboxymethylcellulose for adhesion prevention.

A predisposing factor for the development of jejunal impactions was not identified. Four of the horses (cases 1, 4, 5, and 6) were at a horse show prior to the development of colic requiring exploratory celiotomy. A previous study has concluded there is an association between the development of colic and changes in activity levels, diet, and stabling [56]. All of these management factors are disrupted when horses compete at horse shows, and these factors are similar across all disciplines. Another study has found an association with travel in the previous 24 h and horses diagnosed with simple colonic obstruction and distension [57]. Prevention of jejunal impactions may center on strategies to reduce the risk of impactions in general, including maintaining hydration, and providing consistent feed and exercise.

Limitations of the study include the nature of a retrospective case series, no long-term follow-up of the cases, and the inability to identify horses medically managed for jejunal impactions for comparison. Additional limitations include surgeon-specific variables, as surgery was performed by several surgeons throughout the study period, and small intestinal biopsies were only acquired for one case. Feed analysis, biopsies and microbiome analysis may be warranted in future cases to help elucidate the cause of this type of simple obstruction.

## 5. Conclusions

In conclusion, primary jejunal impactions are intraluminal, obstructive small intestinal lesions that may present similar to other small intestinal lesions. Pre-operative diagnosis remains difficult, and horses present moderately to markedly painful, similar to strangulating obstructions. Exploratory celiotomy presently is required for both diagnosis and therapeutic management. Manual decompression appears sufficient in this small case series to resolve the lesion and small intestinal enterotomy is typically not required for successful. However, impaction size, surgeon experience, and small intestinal viability should be taken into consideration for surgical planning on whether an enterotomy is indicated in future cases is warranted.

## Figures and Tables

**Table 1:** Admission vital parameters, baseline hematology, and peritoneal fluid parameters for cases 1 through 6 are listed in Table 1. Abbreviations: MM: mucus membranes, CRT: capillary refill time, HR: heart rate, RR: respiratory rate, Temp: temperature, PCV: packed cell volume, TS: total solids. # case number.

Case #	1	2	3	4	5	6
HR (bpm)	52 bpm	64 bpm	60 bpm	64 bpm	44 bpm	50 bpm
RR (brpm)	22 brpm	18 brpm	28 brpm	20 brpm	12 brpm	20 brpm
Temp (°F)	98.5 °F	99.9 °F	100.5 °F	100.1 °F	99.5 °F	99.7 °F
Borborygmi	Decreased all quadrants	Decreased on left, normal on right	Decreased on left, normal on right	Gas/fluidy in L dorsal quadrant, absent elsewhere	Decreased all quadrants	Decreased all quadrants
MM (Color)	Pink, moist	Pink, tacky	Pink, moist	Dark pink, tacky	Pale pink, tacky	Pale pink, dry
CRT (s)	<2 s	3 s	<2 s	3–4 s	2–3 s	>3 s
pH	7.43	7.42	7.47	7.37	7.38	7.38
Lactate (mmol/L)	0.9 mmol/L	7.8 mmol/L	2.4 mmol/L	3.1 mmol/L	2.3 mmol/L	1.0 mmol/L
Na (mmol/L)	137 mmol/L	137 mmol/L	138 mmol/L	139.2 mmol/L	139 mmol/L	137 mmol/L
Cl (mmol/L)	102 mmol/L	101 mmol/L	102 mmol/L	97.5 mmol/L	102 mmol/L	100 mmol/L
K (mmol/L)	3.0 mmol/L	3.4 mmol/L	3.4 mmol/L	3.32 mmol/L	3.4 mmol/L	4.1 mmol/L
Ca (mmol/L)	5.7 mmol/L	1.35 mmol/L	5.1 mg/dL	5.5 mg/dL	5.7 mg/gL	5.8 mg/dL
Mg (mg/dL)	0.9 mg/dL	0.9 mg/dL	0.7 mg/dL	1.2 mg/dL	1.0 mg/dL	1.2 mg/dL
Cr (mg/dL)	1.1 mg/dL	1.6 mg/dL	1.7 mg/dL	1.8 mg/dL	1.3 mg/dL	1.2 mg/dL
BUN (mg/dL)	13 mg/dL	20 mg/dL	19 mg/dL	20 mg/dL	17 mg/dL	15 mg/dL
PCV (%)	43%	37%	42%	55%	37%	33%
TS (g/dL)	6.5 g/dL	7.1 g/dL	7.3 g/dL	9 g/dL	5.5 g/dL	7.1 g/dL
Abdominocentesis Color	Clear yellow	Dark yellow	Dark yellow	Dark yellow	Clear yellow	Bright yellow
Abdominal Lactate (mmol/L)	2.6 mmol/L	12.7 mmol/L	7.3 mmol/L	3.2 mmol/L	2.6 mmol/l	3.1 mmol/L
Abdominal TS (g/dL)	<2.0 g/dL	2.8 g/dL	2.6 g/dL	4.0 g/dL	<2.0 g/dL	2.6 g/dL

## Data Availability

The original contributions presented in this study are included in the article. Further inquiries can be directed to the corresponding author.

## Abbreviations

ESGD—Equine Squamous Gastric Disease
